# Impact of Urban-Rural Resident Basic Medical Insurance integration on individual social fairness perceptions: evidence from rural China

**DOI:** 10.3389/fpubh.2024.1408146

**Published:** 2024-08-29

**Authors:** Dandan Liu, Yifei Chu

**Affiliations:** ^1^Institute of Finance and Economics, Shanghai University of Finance and Economics, Shanghai, China; ^2^Institute of Agricultural Science and Technology Information, Shanghai Academy of Agricultural Sciences, Shanghai, China

**Keywords:** equal opportunities, social security, Urban-Rural Resident Basic Medical Insurance (URRBMI), social fairness perception (SFP), rural China

## Abstract

**Background:**

Achieving universal health insurance coverage has become a fundamental policy for improving the accessibility and equity of healthcare services. China's Urban-Rural Resident Basic Medical Insurance (URRBMI) is a crucial component of the social security system, aimed at promoting social equity and enhancing public welfare. However, the effectiveness of this policy in improving rural residents' social fairness perceptions (SFP) remains to be tested.

**Objective:**

To examine the impact of the urban-rural resident basic medical insurance (URRBMI) on rural residents' social fairness perception (SFP) in China.

**Methods and samples:**

The study utilizes city-level and national micro-survey (CGSS) datasets, applying a time-varying difference-in-difference (DID) approach to analyze the equity effects of URRBMI. Excluding urban samples, the final dataset consists of 20,800 rural respondents from 2010, 2011, 2013, and 2015, covering 89 cities.

**Results:**

Key findings reveal that URRBMI has a significant negative effect on SFP. The impact varies depending on the integration model and intensifies over time. Additionally, the negative effect shows heterogeneity based on income, age, health, and region.

**Conclusion:**

This study highlights the complexities and impacts of integrating China's urban and rural healthcare systems. It provides a detailed understanding of the role of URRBMI in rural China, emphasizing the need for targeted approaches to improve rural residents' perceptions of social fairness. The research offers specific policy recommendations, such as establishing differentiated contribution standards, implementing welfare policies favoring rural residents, and adopting varied reimbursement rates for different diseases.

## 1 Introduction

Comprehensive health insurance coverage has become a cornerstone policy globally, aimed at improving the availability and equity of healthcare services (1–4). As the world's largest developing country, China has achieved the landmark feat of establishing a nationwide health insurance system in 2011 on a scale unparalleled in the history of global healthcare (5). Even though there is a significant expansion in societal health insurance protection during the early 2000s, China is still grappling with the challenges of persistent inefficiencies and imbalances in its healthcare system (6–8). Historically, China's Health Insurance System has been divided into the Urban Residents' Basic Medical Insurance (URBMI) for urban residents and the New Rural Cooperative Medical Scheme (NRCMS) for rural residents. This dual-structured framework in a notable disparity in healthcare benefits between urban and rural residents (9, 10), with urban residents enjoying more privileges in terms of reimbursement, healthcare accessibility, and resource allocation (9, 11, 12). An unfair healthcare system not only perpetuates but also exacerbates the socio-economic divide, severely undermining the overall welfare of rural residents. This inequality transcends material wellbeing and profoundly affects residents' subjective perceptions of social fairness.

Individual social fairness perception (SFP) reflects the equitable outcome of resource distribution and plays a critical role in maintaining social stability (13, 14). Many developed countries, intergovernmental organizations, and scholars consider subjective national wellbeing, such as SFP, as an indicator of social progress (15, 16). Pursuing equality or fairness is a major goal of healthcare systems worldwide (2, 17). Individual SFP is closely linked to a well-functioning and just medical insurance system, prompting governments to reform the existing health insurance model. To promote urban-rural equity and eliminate disparities in medical insurance benefits, the State Council integrated URBMI and NRCMS in 2016, forming the Urban-rural Resident Basic Medical Insurance (URRBMI). Overcoming the disadvantages of the urban-rural fragmented urban-rural healthcare system, especially regarding payment and reimbursement processes, depended on large-scale fiscal investments (5, 18). According to China's 2020 national fiscal data, over 31% of health finance expenditures were allocated to subsidize the basic medical insurance fund. The URRBMI policy, aimed at equalizing health services, is crucial for improving medical service opportunities for rural residents and strengthening economic protection, and has been proven to make substantial progress in enhancing welfare equity for lower socio-economic groups (9, 19).

Two streams of literature are relevant to this study: the formation of SFP and the equity of benefits under URRBMI. Extensive studies have developed a comprehensive framework to explain the complex factors affecting social fairness perceptions. Mainstream determinants identified include institutional elements such as democratic participation (20), policy trust (21), and strategies related to social security and income distribution (22). Additionally, some researchers assert that the formation of SFP is intrinsically linked to an individual's social class from a sociological structural perspective. Additionally, researchers assert that the formation of SFP is intrinsically linked to an individual's social class from a sociological structural perspective. This dynamic manifests a dichotomy with vested interests advocating for the maintenance of existing distributional mechanisms. In contrast, disadvantaged groups are inclined toward a fair distributional model rooted in the 'principle of equality (23, 24). Furthermore, some academics argue that fairness judgments are not solely based on the absolute value of acquired benefits but are significantly influenced by relative, comparative outcomes. This concept is known as “relative deprivation theory”(25, 26). A wealth of empirical studies have verified that comparisons with a reference group affect individuals' subjective wellbeing (27, 28).

Concerning the equity of benefits under URRBMI, existing research debates whether URRBMI encourages medical resources and insurance reimbursements to favor disadvantaged groups. The majority of the studies conclude that URRBMI has unified the payment standards and reimbursement benefits for urban and rural residents at the system design level, alleviating the health inequalities perpetuated by systemic stratification (9, 11, 12). The primary beneficiaries of this policy shift are the socioeconomically disadvantaged groups, particularly those with lower income and poorer health conditions, who have seen a significant uplift in their access to healthcare (9, 29, 30). Conversely, a segment of scholars contend that the merging health insurance policies could inadvertently intensify disparities in accessing healthcare services (31, 32). They argue that the benefits of insurance are actualized through the purchase of medical services. Wealthier individuals, with their greater financial resources, are capable of accessing a broader range of higher-quality medical services, resulting in more substantial health insurance reimbursements and protections. This situation ultimately leads to “reverse subsidization”, where the economically disadvantaged inadvertently end up subsidizing the more affluent (32, 33).

Existing research has made significant progress in addressing the welfare inequities under China's URRBMI system, examining factors such as consumption, medical service utilization, and poverty alleviation. However, an important question remains: does the URRBMI policy, aimed at equal opportunity, enhance the SFP of rural residents? There is a substantial gap in the literature concerning the impact of this system on subjective fairness perceptions, particularly from the perspective of relative deprivation. Field surveys reveal that despite the positive impact of URRBMI on healthcare conditions for farmers, a paradoxical sentiment remains among some individuals, who perceive NRCMS as more beneficial. It remains to be answered whether the URRBMI system can successfully uphold the successes of prior reforms and authentically boost the SFP among rural residents. Our examination of China's health insurance integration process not only provides clarity on the Chinese model and its mechanisms but also holds wide-reaching implications for the formulation of healthcare and welfare policies in other nations with similar development situations.

This paper aims to delve into the impact of equal opportunity in healthcare services on SFP based on the URRBMI institution, combining the dimensions of “vertical sense of fairness” and “horizontal sense of fairness”. Through quantitative analysis, this study attempts to answer a crucial question: whether a basic public service system based on “equal opportunity” can effectively enhance residents' perception of fairness and function as a “social stabilizer”. Compared to prior studies, this paper offers three notable contributions: Firstly, existing literature on the impact of URRBMI primarily focuses on health equity and income equality. This study concentrates on the effects of medical resource allocation on individuals' subjective perceptions, thereby extending the discussion scope of the urban-rural integrated medical insurance system. Secondly, equal opportunity is the core of the URRBMI, and SFP reflects rural residents' subjective views on urban-rural equity. The effectiveness of the “equal opportunity” approach in medical resource allocation in enhancing rural residents' SPF warrants further examination. This paper examines the relationship between the equalization of basic public service opportunities and the sense of fairness among rural residents. Lastly, few studies have utilized the China General Social Survey (CGSS) database for policy effect evaluation using a difference-in-differences(DID) model. This is primarily due to the CGSS database not providing direct administrative codes or names for the cities of the samples, making it challenging to identify the regions where policies were implemented. This study addresses this limitation by using respondents' detailed birthplace information (city, county/district) and matching it with samples where rural residents' birthplace and current location are consistent. By inferring the corresponding prefecture-level city codes and names, this approach enables the combination of nationwide micro-survey data with the URRBMI policy, thereby expanding the application scope of CGSS data.

The subsequent parts of this document are structured as follows: Section 2 reviews the institutional background of URRBMI and outlines the theoretical framework, Section 3 introduces the econometric methods and describes the datasets involved in the study, Section 4 reports the empirical results, and Section 5 distills the research conclusions and proposes policy recommendations.

## 2 Background and theoretical framework

### 2.1 Institutional background

#### 2.1.1 Background of the integration of URRBMI

Since the beginning of the 21st century, China has successfully established a basic healthcare system covering the vast majority of urban and rural residents, with the Urban Resident Basic Medical Insurance (URBMI) and the New Rural Cooperative Medical Scheme (NRCMS) being its integral components (34). By 2016, the coverage of these two systems had exceeded 95%. URBMI and NRCMS operate on a strictly differentiated basis according to household registration. The NRCMS is a health insurance policy targeting the rural population, which has been implemented since 2003. NRCMS has effectively served as a commendable policy in providing basic medical security for Chinese farmers. However, significant disparities in medical benefits between residents living in urban and rural areas have perpetuated longstanding health inequalities in rural areas.

To mitigate these disparities, in 2016, the State Council published the document entitled “Opinions on Integrating Urban-Rural Resident Basic Medical Insurance Systems”. Building upon the experiences of URBMI and NRCMS, the URRBMI was established, characterized by the implementation of a “six-unification” standard, encompassing uniform coverage, financing policy, security treatment, medical insurance catalog, designated management, and fund management, thereby achieving equal opportunity in the utilization of medical insurance benefits.

Regional variations in the institutional design of integrated urban and rural medical insurance primarily manifest in two models: “one system, one standard” and “one system, multiple standards”. The former adopts a unified standard for financing and treatment design, while the latter implements differentiated financing and treatment levels within a unified policy framework. The “one system, one standard” design implements a unified and singular standard for funding and benefits across urban and rural areas. For instance, Guangzhou's 2015 guidelines state that the individual contribution for urban and rural residents' medical insurance is 152 RMB per person, with uniform contributions entitling residents to identical benefits. Conversely, the “one system, multiple standards” design, while maintaining a unified policy framework, employs differentiated funding and benefit levels based on the “more pay, more gain” principle, allowing residents to choose their contribution level according to their needs. For example, Luzhou's 2015 contribution standards for urban and rural adult residents' medical insurance offer a low tier (90 RMB/person) and a high tier (220 RMB/person), with residents selecting their preferred level. Correspondingly, inpatient medical benefits are adjusted according to the contribution level, with two different reimbursement rates for covered medical expenses, reflecting the principle of proportional rights and obligations.

In addition, we collect and organize policy documents from various regions and find that there are differences in the implementation time of the URRBMI institutions in different cities. Chengdu, the earliest city to integrate urban and rural residents' medical insurance, completed the integration of urban resident insurance and the new rural cooperative medical scheme as early as 2009. In contrast, some areas, such as Nanjing, did not complete the integration until 2019. This gradual implementation offers a unique quasi-natural experimental condition for an in-depth exploration of the relationship between URRBMI and rural residents' SFP using a progressive difference-in-differences model. Among the cities involved in the study period, 20 cities adopted different financing and governance models based on their specific circumstances at various times^1^.

#### 2.1.2 Transition from NRCMS to URRBMI

During the integration process, accounts from NRCMS and URBMI merged into a unified URRBMI account, achieving integrated management of medical insurance fund expenditures for both urban and rural areas (Table 1). Additionally, the structure of medical insurance accounts and the reimbursement ratios underwent significant changes. During the NRCMS period, independent personal medical fund accounts were established to increase farmers' willingness to enroll, with individual insurance premiums deposited into these accounts for covering out-of-pocket expenses for medications, outpatient, and hospitalization services. Under URRBMI, personal medical fund accounts were gradually phased out, consolidating individual payments and government subsidies into a social pooling account. In terms of reimbursement ratios, unlike the centralized coordination during the NRCMS period, regions have the autonomy to set their reimbursement ratios for residents' health insurance during the URRBMI period. Notably, there are obvious differences in the reimbursement rates across regions, possibly reflecting differential considerations of local healthcare needs and financial capacity.

**Table 1:** Characteristics of NRCMS and URRBMI Insurance Systems.

	**NRCMS**	**URRBMI**
Target population	Rural residents	Urban and rural residents
Voluntary enrollment	Yes	Yes
Individual contribution	Yes	Yes
Government subsidy	Yes	Yes
Enrollment unit	Household	Household
Reimbursement ratio	Outpatient: 20%−50%	Outpatient: 40%−50%
	Hospitalization: 30%−60%	Hospitalization: 55%−90%
Integration level	County	City
Fund management	DREMS	FBFAMS; SFA; DREMS

In the table, FBFAMS, Fund Budgeting and Final Accounting Management System; SFA, Special Fiscal Account, and DREMS represents Dual Revenue and Expenditure Management System.

### 2.2 Theoretical framework

As a measure to integrate urban-rural health insurance in China, URRBMI has indeed made significant progress in equalizing the medical treatment and resource disparity between urban and rural residents (6, 12). The increase in reimbursement rates and the expansion of medical service coverage are indicative of a substantial reduction in the inequality of healthcare resource utilization among these populations. For instance, the policy has elevated reimbursement for certain chronic illnesses and major medical expenses, enhancing the affordability of healthcare for rural inhabitants. However, there persists a contradictory sentiment among some rural individuals, who perceive URRBMI as not contributing to an increased sense of fairness. To explore whether URRBMI improves farmers' SFP, we have developed a theoretical analysis and hypotheses.

The SFP of rural residents is shaped by both longitudinal (LF) and horizontal fairness (HF) perceptions (14, 35). LF perception emerges as farmers compare the benefits received under URRBMI with those from the NRCMS period. The formation of HF occurs when the farmer compares himself with urban residents or others who enjoy similar benefits. With the blurring of the urban-rural boundary and the widespread use of digital technology in rural areas, farmers' access to information has been greatly broadened, which has changed their point of reference in assessing SFP (14, 15, 36). Grounded in the conceptual frameworks of LF and HF, this study examines the mechanisms of URRBMI affecting farmers' SFP with a focus on premium payment and reimbursement.

Firstly, we analyze the impact of URRBMI on rural residents' SFP from the perspective of payment reform. An analysis of historical data reveals a progressive increase in the personal contribution requirements for URRBMI^2^. Since 2018, individual insurance expenses have constituted over 1.5% of the average disposable income of rural residents, indicating a relative increase in their financial burden compared to the NRCMS period. On the other hand, since urban-rural populations have been included in the same insurance system and are subject to the same contribution standards, the vast group of low-income rural residents have taken on more responsibility for health insurance financing than they did before. This inadvertently creates the trap of “exploitation of low-income by high-income earners”. Hence, post-integration, rural residents potentially experience a subjective sense of loss in terms of premium payments, both in longitudinal comparison with the NRCMS period and in horizontal comparison with urban residents.

Secondly, we analyze the impact of URRBMI on rural residents' SFP from the perspective of reimbursement reform. Although there has been a nominal increase in the reimbursement ratio for rural residents under URRBMI compared to the NRCMS period, the actual perceived reimbursement ratio has not met the farmers' expectations. Medical expense payments in China involve a co-payment scheme between medical insurance funds and residents. Under the co-payment reimbursement system of URRBMI, the allocation of medical resources and payment is tightly linked to income levels. Given the generally lower income of farmers, who also need to set aside funds for future agricultural production, their real disposable income is considerably lower, resulting in a higher medical burden compared to urban residents. Furthermore, the distribution of healthcare resources across China shows a marked difference between urban and rural areas, as premium healthcare facilities are mainly found in major cities and their hospitals. This disparity leads to higher transportation costs and labor costs for farmers to access healthcare. The unified urban-rural medical insurance achieves only “formal equality of opportunity” in reimbursement standards, failing to perform the fundamental role of income redistribution in social security. The above reasons have resulted in a permanent disadvantage of “relative deprivation” of the rural population. Drawing from the preceding analysis, we formulate Hypothesis I.

Hypothesis I::URRBMI will reduce rural farmers' subjective SFP.

Based on the above analysis, we find that the decrease in farmers' SFP is more likely to derive from the loss of “horizontal access” through the positioning of urban residents as reference points. In terms of URRBMI's implementation, it is categorized into two models: “one system, one standard” and “one system, multiple standards”. The central government's setting of the minimum standards for URRBMI means that the former approach does not significantly alter the premium burden or actual reimbursement ratio for rural residents. Conversely, the latter model, which involves differentiated funding and benefit levels within a unified policy framework, may be the fundamental cause of the reduced SFP among farmers. In the current context of significant income disparity between urban and rural residents, urban dwellers are more likely to choose higher payment tiers. Following the principle of “more pay, more benefits”, those in higher tiers receive greater reimbursement ratios, leading to a widened gap in reimbursement between tiers and further intensifying the “inverse distribution” of medical resources. We propose Hypothesis II.

Hypothesis II: The impact of differentiated integration models on rural residents' SFP varies, with the multi-standard URRBMI model exerting a more depressive effect.

Assuming that Hypothesis I and Hypothesis II are valid, URRBMI leads to lower SFP among rural residents. They believe that urban residents have access to more healthcare resources. Under this premise, urban residents benefit from enhanced health human capital, which in turn elevates personal and family productivity, and improves their income conditions (37). This leads to urban families continually accessing greater medical resources, creating a “virtuous cycle”. In contrast, the situation for farmer families is the opposite. As the income disparity between urban-rural residents increases, the unequal distribution of medical resources between these groups may become more severe than it was at the beginning of the integration. We propose Hypothesis III.

Hypothesis III: The negative effect of URRBMI on the rural residents' SFP will increase over time.

## 3 Data and method

### 3.1 Datasets

This study utilizes both macroeconomic statistical data and micro-level survey data. The macroeconomic data is sourced from the “China Urban Statistical Yearbook”, “China Regional Economic Statistical Yearbook”, “China Health Statistical Yearbook”, and other relative statistical yearbooks. The micro-level data is derived from the China General Social Survey (CGSS) provided by the China Survey and Data Center at Renmin University of China. This data encompasses information on Chinese society, communities, families, and individuals involving adult citizens aged over 18 years. Given that the NRCMS had essentially achieved full rural coverage by 2010, this study has retained sample data from 2010 onwards.

Due to privacy protection considerations, the CGSS database ceased to provide codes for survey regions after the year 2015. Consequently, this study utilizes data up until 2015, which includes codes for regional samples. It is important to note that the CGSS data do not directly reveal the prefecture-level city of respondents but only display codes for their provinces and municipalities. Therefore, this research retains samples where the birthplaces of rural residents coincide with their current locations, and employs respondents' detailed addresses of their birthplaces (including cities and districts) to deduce the codes corresponding to each prefecture-level city. Excluding urban samples, the final dataset comprises 20,800 rural respondents from the years 2010, 2011, 2013, and 2015, spanning 31 provinces and 89 cities. Among the surveyed cities in the CGSS, 20 cities, including Chongqing and Chengdu, implemented the integration of urban and rural resident medical insurance before 2016. During the integration process, each city adopted different funding and reimbursement models based on their specific circumstances. Detailed information is provided in Supplementary Table S1.

### 3.2 Models and variables

In this study, mixed cross-sectional data are used to construct the asymptotic difference (DID) model. The specific model is constructed as follows (Equation 1):


(1) $$\begin{array}{l} Equ_{cit}=\alpha +\beta Treat_{c}\times Post_{ct}+X_{it}^{\prime }\chi +Y_{ct}^{\prime }\delta +\mu_{c}+\mu_{t}+\varepsilon_{cit} \end{array}$$

The explanatory variable *Equ*_*cit*_ represents the SFP of rural resident *i* of city *c* in time period *t*. The SFP is measured using the question “In general, do you think that today's society is fair”. Based on respondents' answers, ratings of “not at all fair”, “somewhat unfair”, “neutral”, “somewhat fair”, and “completely fair” are assigned numeric scores of 1, 2, 3, 4, and 5, forming an ordinal variable.

*Treat*_*c*_ is utilized to identify cities implementing URRBMI. If a city has implemented URRBMI, it is assigned a value of 1, otherwise, it is 0. *Post*_*ct*_ is the timing of URRBMI, assigning a value of 0 to years before integration and 1 to the year of integration and thereafter. We ultimately obtained 2,606 samples for the treatment group and 18,194 samples for the control group. μ_*c*_ represents regional fixed effects; μ_*t*_ denotes year fixed effects; ε_*cit*_ is a random disturbance term; α and β are parameters to be estimated; χ and δ are vectors of parameters to be estimated.

This article incorporates several control variables (X) as follows: Gender (1 for males, 0 for females), Age , Education level (assigned values from 1 to 13 based on the highest education level), Political affiliation (1 for Communist Party or democratic party members, 0 otherwise), Marital status (1 for cohabiting, first marriage, or remarried with spouse, 0 for others), Health level (values from 1 to 5), Employment type (1 for non-agricultural employment, 0 otherwise), Personal income (natural logarithm of total personal income last year), and Digital literacy (1 for frequent media use, 0 otherwise). Descriptive statistics of these variables are presented in Table 2.

**Table 2:** Descriptive statistics of variables.

	**Full sample**		**Treatment group**		**Control group**	
**Variables**	**Mean**	**SD**	**Mean**	**SD**	**Mean**	**SD**
SFP	3.151	1.050	3.226	1.009	3.140	1.055
Gender	0.474	0.499	0.473	0.499	0.474	0.499
Age	48.324	15.891	48.358	16.456	48.319	15.806
Marriage	0.821	0.383	0.804	0.397	0.824	0.381
Party	0.047	0.211	0.045	0.208	0.047	0.212
Education	3.555	2.035	3.529	2.248	3.558	2.002
Health	3.463	1.187	3.387	1.156	3.473	1.190
Employment	0.291	0.454	0.304	0.046	0.289	0.454
Income	8.349	3.970	8.267	4.011	8.361	3.964
Digital	0.149	0.356	0.189	0.392	0.1433	0.351

## 4 Empirical results

### 4.1 Benchmark regression results

This study employs a two-way fixed effects regression, with the results presented in Table 3. The regression in Column (1) indicates a significant negative impact of URRBMI on rural residents' SFP. We add individual-level and city-level control variables in Columns (2) and (3) in turn, and the results are consistent with Column (1). Specifically, URRBMI leads to a 10.1% decrease in rural residents' SFP according to the results in Column (3). Hypothesis I is verified.

**Table 3:** Benchmark regression results.

	**(1)**	**(2)**	**(3)**	**(4)**
**Variables**	**SFP**	**SFP**	**SFP**	**SFP**
*Treat*_*c*_ × *Post*_*ct*_	−0.127^***^	−0.107^**^	−0.101^**^	-
	(0.044)	(0.042)	(0.041)	
DID (one-standard model)	-	-	-	−0.067
				(0.047)
DID (multi-standard model)	-	-	-	−0.194^***^
				(0.056)
Individual-level control variables	-	Yes	Yes	Yes
City-level control variables	-	-	Yes	Yes
Year-fixed effect	Yes	Yes	Yes	Yes
City-fixed effect	Yes	Yes	Yes	Yes
N	20,800	20,800	20,800	20,800
R^2^	0.028	0.054	0.055	0.055

(1) ^**^ and ^***^ Denote significant at the 5% and 1% levels. (2) Standard errors in parentheses are clustered at the township level.

To further discuss the impact of different integration models on rural residents' SFP, we identify cities with “one system, one standard” and cities with “one system, multiple standards”. The regression results are shown in Column (4). The URRBMI of the multi-standard model has a stronger suppressive effect on the SFP of rural residents, implying that this type of healthcare integration design is less conducive to the enhancement of rural residents' sense of social equity. The reason may be that under the “more pay, more benefits” principle, higher contributions entail higher reimbursement ratios and subsidies. For higher-income urban residents, the cost difference between high and low tiers is minimal relative to their disposable income, making them more likely to pay higher premiums. This results in urban residents receiving higher medical subsidies compared to rural residents at lower tiers. Consequently, low-income farmer groups are disadvantaged in the distribution of medical insurance benefits, exacerbating negative perceptions of social fairness among rural residents. Hypothesis II is verified.

### 4.2 Identification condition test of DID

To obtain an unbiased estimate of the policy variable's Difference-in-Differences (DID) coefficient β, it is essential that this variable is uncorrelated with the random disturbance term ε_*cit*_, fulfilling *cov*(*Treat*_*c*_, ε_*cit*_) = 0 and also satisfying *cov*(*Post*_*ct*_, ε_*cit*_) = 0. Therefore, this paper needs to address two key issues: the randomness in the selection of cities for urban-rural resident medical insurance integration and the randomness concerning the timing of this integration.

#### 4.2.1 Randomness issue in the selection of cities for URRBMI integration

Due to the absence of specific documents as reference standards for the selection of cities for URRBMI integration, this paper attempts to identify the key determinants influencing the choice of cities for insurance integration. Specifically, considering that URRBMI is coordinated at the city level, the study examines potential determinants from the perspective of city characteristic variables. Following the existing study (38), a Logit model is constructed to estimate the probability of various factors influencing the implementation of URRBMI in a region. The dependent variable is whether a city is designated for integration, with cities under URRBMI integration coded as 1 and others as 0. Factors related to the city's medical development level, such as economic development, urban population size, healthcare financial investment, the proportion of healthcare investment in total fiscal expenditure, the number of hospitals and clinics, and the number of beds in hospitals and clinics, are selected as explanatory variables for whether a city integrates URRBMI. Moreover, as the decision to integrate URRBMI primarily references the city's data from the previous year, explanatory variables are included as one-period lagged terms in the model, with price variables indexed to the base year of 2009.

The estimation results are shown in Table 4. The findings indicate a correlation between the decision to merge medical insurance for urban and rural residents and factors such as the city's economic development level, urban population size, healthcare financial investment and healthcare level (number of hospital beds and the count of medical facilities including hospitals and clinics). Therefore, to control for potential endogeneity in the selection of cities for URRBMI integration, the model should include one-period lag suggestions terms of the aforementioned city characteristic variables. Additionally, the lagged term of the proportion of healthcare expenditure in total fiscal expenditure is also included in the model to mitigate estimation biases caused by omitted variables.

**Table 4:** Identification condition test.

**Variables**	**URRBMI**
Economic development level	0.141^*^
	(0.078)
Urban population size	−0.001^***^
	(0.000)
Healthcare financial investment	−0.314^***^
	(0.099)
Proportion of healthcare investment in fiscal expenditure	0.015
	(0.017)
Number of beds in hospitals and clinics	0.337^**^
	(0.148)
Number of hospitals and clinics	0.002^***^
	(0.001)
*N*	6,620
R^2^	0.3679

Standard errors in parentheses are clustered at the township level. ^*^, ^**^, ^***^ Denote significance at the 10%, 5%, and 1% levels, respectively.

#### 4.2.2 Randomness issue in the timing: parallel trend test

The second condition for the applicability of the Difference-in-Differences (DID) model is the satisfaction of the parallel trend assumption. In this study, this implies that there were no systematic differences in the social fairness perception among rural residents across regions prior to the implementation of URRBMI integration. A parallel trends test model is constructed in this section. The model is as Equation 2.


(2) $$\begin{array}{l} Equ_{cit}=\alpha +\sum \beta_{t}Treat_{c}\times ryear_{ct}\\ \;+X_{it}^{\prime }\chi +Y_{ct}^{\prime }\delta +\mu_{c}+\mu_{t}+\varepsilon_{cit} \end{array}$$

Where, *ryear*_*ct*_ represents relative year dummy variables. Due to the different timings of URRBMI integration across cities, there is a need to establish time variables that account for these relative differences. Consequently, this study employs the widely used event study methodology to conduct this test. Specifically, the year when URRBMI was integrated is labeled as 0, the subsequent year (or n years after) is assigned a value of +1 (+n), and the year prior (or n years before) is given a value of−1 (-n). Other variables and parameters are consistent with Model (1). Following Fajgelbaum et al. (39), due to a smaller number of observations at the extremes, all relative years ≤ -3 are uniformly coded as −3, and all years greater than or equal to +5 are coded as +5, resulting in a year span of [−3,5].

**Figure 1:**
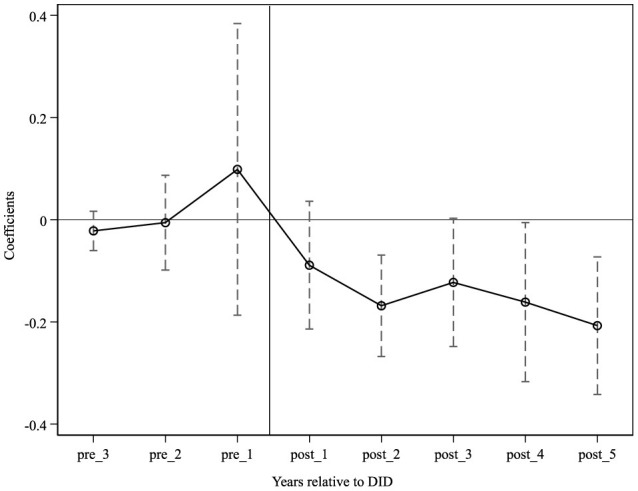
Parallel trend.

For a more intuitive observation of the parallel trend assumption test and the dynamic effects of the integrated medical insurance impact, the 90% confidence interval of the coefficients is illustrated in Figure 1. As indicated in the figure, before the implementation of URRBMI, the regression coefficients are not significant, suggesting that the URRBMI shock does not lead to significant differences in rural residents' SFP. This satisfies the pre-trend assumption. After the integrated medical insurance program, the absolute value of the regression coefficients gradually increases, indicating that the mitigating effect of URRBMI strengthens over time. Hypothesis III is thus validated.

The parallel trends test offers a clearer view of the dynamic effects of URRBMI (Figure 1). It is evident that before the implementation of URRBMI, the regression coefficients are insignificant, indicating that the URRBMI shock does not lead to significant differences in the SFP of rural residents, thus meeting the pre-trend assumption. Post-integration, there is a gradual increase in the absolute value of the regression coefficients, suggesting that the diminishing effect of URRBMI intensifies over time. Hypothesis III is verified.

### 4.3 Robustness test

#### 4.3.1 Adjusting the sample of the experimental group

Considering that the sample period of this paper is ended in 2015, cities that implemented health insurance coordination in the year of 2015 may not be able to show the policy effect instantly. To validate whether the benchmark regression estimates are robust, we run the regression after setting the *Treat*_*c*_ variable to 0 for the cities that implemented URRBMI in 2015. The regression results are shown in Column (1) of Table 5, which shows that the regression results are consistent with the benchmark results.

**Table 5:** Robustness test results.

	**(1)**	**(2)**	**(3)**	**(4)**
**Variables**	**DID**	**PSM-DID**	**Oprobit**	**Probit**
*Treat*_*c*_ × *Post*_*ct*_		−0.163^***^	−0.111^**^	−0.057^***^
		(0.062)	(0.044)	(0.021)
DID_new	−0.155^***^			
	(0.049)			
Individual-level control variables	Yes	Yes	Yes	Yes
City-level control variables	Yes	Yes	Yes	Yes
Year-fixed effect	Yes	Yes	Yes	Yes
City-fixed effect	Yes	Yes	Yes	Yes
N	20,800	4,546	20,800	20,800
R^2^	0.055	0.068	0.022	0.040

^*^, ^**^, ^***^ Denote significance at the 10%, 5%, and 1% levels, respectively.

#### 4.3.2 PSM-DID analysis

**Figure 2:**
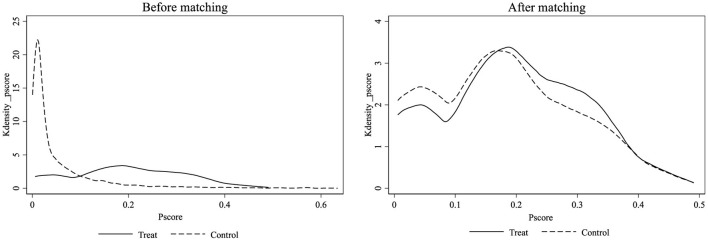
Kernel density.

To minimize the selectivity bias more effectively, this paper further uses the fixed utility model based on propensity matching for estimation. As this paper uses mixed cross-section data, year-by-year matching is used in performing the matching. The kernel density plots before and after matching are shown in Figure 2, and the regression results of the matched samples are shown in Column (2) of Table 5, which shows that there is a consistency between the regression results and the baseline results.

#### 4.3.3 Replacing the estimation model

Firstly, considering SFP is an ordered discrete choice, we conducted robustness checks using an ordered probit model for Model (1). The regression outcomes, as shown in Column (3) of Table 5, are consistent with the baseline regression results. Secondly, acknowledging the data characteristics of SFP, categorical variables may better capture respondents' true sentiments regarding social fairness. We transformed the ordinal variables into binary ones, assigning a value of 0 to responses “completely unfair”, “somewhat unfair”, and “neutral”, and a value of 1 to “somewhat fair” and “completely fair”. The results displayed in Column (4) of Table 5 reaffirm the findings of the baseline regression.

#### 4.3.4 Placebo test

To further examine whether the impact of URRBMI on rural residents' SFP is driven by extraneous random factors, this paper performs placebo tests following the methodology of Cai et al. (40). Given that this study is predicated on a multi-period DID model, the extraction of experimental group samples necessitates both locational randomness and temporal randomness. We randomly select 21 cities out of 89 as the treatment group. A random year is chosen for each city as its policy year, creating a new treatment group with random city and policy time. Model (1) is estimated 1,000 times.

**Figure 3:**
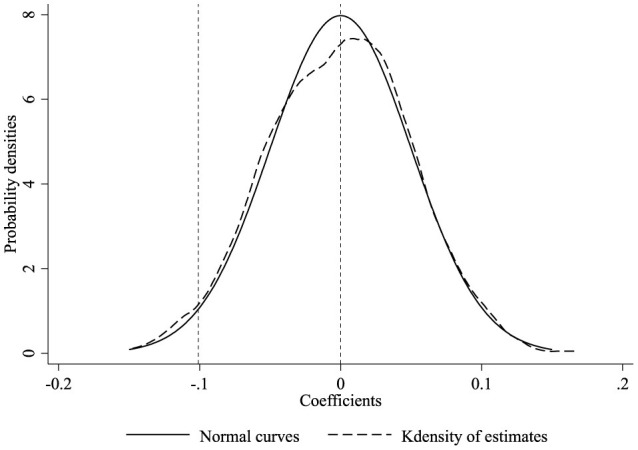
Placebo test. The dashed line represents the kernel density curve of the coefficient estimates; the solid line is a normal distribution curve with a mean of zero.

The distribution of the regression coefficients and probability density are depicted in Figure 3. The kernel density graph of the coefficient estimates nearly coincides with a normal distribution centered at zero, and the 1,000 estimated coefficients yield a mean of −0.0007 and a variance of 0.0502. This indicates that for randomly generated integration cities, no statistically significant inhibitory effect of URRBMI on SFP is observed, confirming the robustness of the benchmark regression.

### 4.4 Heterogeneity analysis

#### 4.4.1 Income heterogeneity analysis

Under the “pay first, reimburse later” scheme of URRBMI, rural residents' income directly affects their access to medical resources. Therefore, we examine the income heterogeneity in URRBMI's impact on rural residents' SFP. We use the poverty line of 2011 as the income threshold, categorizing households with income below this line as poor and others as non-poor. The results for income heterogeneity are shown in Columns (1) and (2) of Table 6. We find that URRBMI does not reduce the SFP of poor households. This is likely linked to China's special medical subsidy policies for poor rural households. To prevent impoverishment due to illness, some regions in China have implemented special medical subsidy policies for people experiencing poverty. For example, in Anhui Province, low-income, poverty-stricken or monitored rural residents receive extra subsidies for their medical expenses. In terms of reimbursement, after deductions through basic medical insurance and major illness insurance, the policy also provides a minimum of 60% assistance for the self-paid portion for those eligible for medical aid. This indicates the enhancement of SFP for groups like poor households still relies on special government subsidies, demonstrating a strong policy dependence for their welfare improvement.

**Table 6:** Heterogeneity analysis.

	**(1)**	**(2)**	**(3)**	**(4)**	**(5)**	**(6)**	**(7)**	**(8)**
**Variables**	**Poor**	**Non-poor**	**Older adults**	**Non-older adults**	**Healthy**	**Unhealthy**	**Western**	**Non-western**
*Treat*_*c*_ × *Post*_*ct*_	−0.078	−0.108^**^	−0.043	−0.128^***^	−0.095^**^	−0.102^*^	−0.154^***^	−0.012
	(0.068)	(0.044)	(0.070)	(0.045)	(0.045)	(0.059)	(0.055)	(0.055)
Individual-level control variables	Yes	Yes	Yes	Yes	Yes	Yes	Yes	Yes
City-level control variables	Yes	Yes	Yes	Yes	Yes	Yes	Yes	Yes
Year-fixed effect	Yes	Yes	Yes	Yes	Yes	Yes	Yes	Yes
City-fixed effect	Yes	Yes	Yes	Yes	Yes	Yes	Yes	Yes
*N*	5,841	14,958	5,468	15,332	11,436	9,364	6,653	14,147
R^2^	0.067	0.045	0.042	0.033	0.060	0.053	0.062	0.050

^*^, ^**^, ^***^ Denote significance at the 10%, 5%, and 1% levels, respectively.

#### 4.4.2 Age heterogeneity analysis

To investigate whether rural older adults have benefited from this round of medical insurance reform, thereby enhancing their perception of social fairness, this study categorizes sample households into older adults and non-older adults groups, examining the differential impact of URRBMI on the SFP of these two categories. Following the social security system's classification, which distinguishes between the older adults and non-older adults using the age threshold of 60 years, respondents aged 60 and above are defined as older adults, while others are considered non-older adults. The results, as presented in Columns (3) and (4) of Table 6, indicate that URRBMI integration mainly decreases the SFP among rural residents below 60 years of age, with no significant impact on residents aged 60 and above. The notable difference could be attributed to the fact that younger people are the primary users of the Internet. The advancements in network technology, promoting interconnectivity, have had a more profound effect on rural youth, altering their reference points for horizontal comparisons. Consequently, rural youth, who find themselves at a disadvantage in comparison with urban residents, are likely to experience a reduced SFP.

#### 4.4.3 Health heterogeneity analysis

The health status of individuals significantly influences their access to and utilization of medical resources, making the analysis of health heterogeneity crucial for a comprehensive understanding of the impact of URRBMI on rural residents' SFP. We use self-rated health as a basis for measuring respondents' health levels, dividing the sample households into healthy and unhealthy groups. The regression results for health heterogeneity, as shown in Columns (5) and (6) of Table 6, indicate that URRBMI's diminishing effect on SFP occurs in both groups. During the NRCMS period, if a farmer had no medical insurance reimbursement in a payment year, the self-contributed portion in their personal account could be used for purchasing medicines at pharmacies. After the cancellation of personal accounts, for healthy farmers, the self-contributed portion becomes entirely a cost for risk transfer, leading to a decrease in their sense of benefit. For farmers with poorer health, this reduction in SFP may stem from greater healthcare needs, higher healthcare costs, and lower incomes associated with lower human resource levels.

#### 4.4.4 Region heterogeneity analysis

In China, medical resource distribution varies greatly across regions. The effectiveness of resident medical insurance is highly dependent on regional healthcare levels. This necessitates an examination of the varied impact of URRBMI on rural residents' SFP across different areas. Considering that there is a remarkable disparity between the level of medical care in the western region and the other regions, this study categorizes the sample into western and non-western areas for analysis. The regression results for this regional heterogeneity, detailed in Columns (7) and (8) of Table 6, reveal that the negative impact on rural residents' SFP is more pronounced in the western region. This disparity indicates that in regions with uneven medical resource distribution, rural residents in areas lacking medical resources struggle more to benefit from the integrated medical care system.

### 4.5 Further analysis

In the previous section, we discover that URRBMI has a suppressive effect on the SFP of rural residents. This finding prompts us to ponder the broader implications of URRBMI on other subjective perceptions held by rural residents. In this part, our focus shifts from the impact of URRBMI on rural residents' subjective SFP to its effects on other dimensions of residents' subjective experiences. This encompasses their perceptions of the fairness of income distribution, their satisfaction with public healthcare services, and their satisfaction with the allocation of public services between urban-rural populations. Income distribution fairness perception (IDFP) is derived from respondents' views on income disparities, with responses to “Some people earn more, some less, do you think this is fair?” rated from 1 to 5, ranging from “strongly disagree” to “strongly agree”. Public healthcare services perception (HSP) measures satisfaction with government-provided medical services, with responses from “very dissatisfied” to “very satisfied” also rated 1 to 5. Public healthcare resources distribution satisfaction (HRDS) assesses views on the equity of public service distribution, rated similarly from 1 to 5 based on satisfaction levels.

The regression results are shown in Table 7. The results show that after the implementation of the URRBMI integration, rural residents' healthcare services perception (HSP) and healthcare resources distribution satisfaction (HRDS) decreased significantly. The findings suggest that although the URRBMI is intended to integrate the rural and urban healthcare systems, its implementation may not fully address underlying disparities, especially subjective inequalities. The gap between policy goals and actual outcomes suggests that truly improving the subjective sense of equity among rural residents will require complementary government measures in other dimensions. For example, combining social welfare programs with health care policies to address wider socio-economic inequalities to improve the overall life satisfaction of rural residents.

**Table 7:** Further analysis results.

	**(1)**	**(2)**	**(3)**
**Variables**	**IDFP**	**HSP**	**HRDS**
*Treat*_*c*_ × *Post*_*ct*_	0.011	−0.104^***^	−0.116^***^
	(0.041)	(0.039)	(0.043)
Individual-level control variables	Yes	Yes	Yes
City-level control variables	Yes	Yes	Yes
Year-fixed effect	Yes	Yes	Yes
City-fixed effect	Yes	Yes	Yes
N	20,800	3,970	20,800
R^2^	0.031	0.057	0.031

^*^, ^**^, ^***^ Denote significance at the 10%, 5%, and 1% levels, respectively.

## 5 Conclusions and policy implications

Promoting social equity and people's wellbeing is the ultimate destination of high quality development of social security. As an essential component of China's social security system, URRBMI assumes the responsibility of promoting the redistribution of healthcare resources and maintaining social equity. We innovatively combine city-level data with large-scale national micro-survey data (CGSS) and conduct a quasi-natural experiment based on the asymptotic implementation of the URRBMI using the time-varying difference (DID) method. The study finds that: (1) URRBMI has a significant negative impact on rural residents' SFP. (2) The impact of differentiated integration models on rural residents' SFP varies, with the multi-standard URRBMI model exerting a more depressive effect. (3) The negative effect of URRBMI on the rural residents' SFP will increase over time. (4) The effect of URRBMI on the SFP of rural residents is heterogeneous according to the income, age, health, and region of the rural household. Specifically, URRBMI has a significant negative effect on the non-poor, the non-older adults, the western region, and the healthy and non-healthy, while it does not have a significant effect on the SFP of the poor, the older adults, and the rural residents in the non-western region.

The findings of this study reveal that although the equalization medical insurance policy, designed with “fairness” in mind, aims to integrate rural and urban healthcare systems and address the inequality in medical resource utilization, relying solely on the expansion of the URRBMI with uniform contribution models and reimbursement rates is insufficient for achieving urban-rural equity. This singular approach has proved to be ineffective in enhancing rural residents' perceptions of social fairness. The continuous revision and reform of social security systems represent a progressive journey toward achieving fairness. To genuinely improve the subjective fairness perceptions of rural residents, the URRBMI policy must be combined with supplementary government measures. Based on the study's findings, the following policy recommendations are proposed:

Firstly, establish differentiated contribution standards based on actual income levels across regions and between urban and rural areas. This involves two key aspects: (1) Adjust the contribution standards for rural residents according to regional development conditions. On one hand, lower the personal contribution standards for rural residents in less developed areas to mitigate the trend of policy dropouts, safeguard the basic medical rights of low-income rural residents, and reduce the risk of poverty due to illness. On the other hand, use central or local government subsidies to compensate for the reduced contributions from rural residents, ensuring the total amount of the medical insurance fund in underdeveloped areas remains stable and preventing a decrease in local residents' medical benefits. (2) Adjust individual contribution standards based on rural residents' income levels. According to the income disparity between urban and rural residents, appropriately reduce the contribution standards for rural residents while increasing the contribution standards for urban residents. This approach balances the actual payment burden, maintains the consistency of contribution levels between urban and rural areas, and ensures the stability of the local medical insurance fund.

Secondly, adjust the reimbursement system to favor rural areas and residents. This includes gradually adding appropriate healthcare services for rural areas to the medical insurance reimbursement catalog, such as allocating part of the medical insurance fund to increase free physical examination programs for rural residents and gradually increasing the proportion of medical insurance funds used for rural healthcare institutions. Additionally, the government can allocate a portion of the medical insurance funds to support village and township health clinics in providing medication delivery services for chronic diseases, simplifying the process of obtaining medications for chronic illnesses and reducing transportation costs for rural residents. As the incidence of chronic diseases rises in rural areas, frequent trips to medical institutions impose high transportation and time costs on rural residents, especially those in remote areas^3^. Establishing a medication delivery service through village clinics can assist rural residents in more conveniently obtaining their required medications.

Lastly, implement differentiated reimbursement rates for various diseases, with a focus on common diseases among rural residents and those with low-income levels. This involves setting disease-specific reimbursement methods based on the severity of diseases, clinical treatment processes, and other characteristics in different regions. The government should set varying reimbursement standards based on disease incidence rates in different areas, raising reimbursement levels in regions with higher rates. Additionally, the reimbursement rate for common and chronic diseases at primary healthcare institutions should be increased. The treatment processes for these conditions are well-established, and the differences in treatment outcomes across healthcare levels are minimal. Directing patients with common and chronic diseases to primary healthcare facilities will reduce the regional financial burden and alleviate the pressure on top-tier medical institutions. This approach ensures that the medical insurance system effectively facilitates patient triage.

## Data Availability

The data used in this study is subject to the following licenses/restrictions: The Chinese General Social Survey (CGSS) data is a significant resource for studying Chinese society and is widely utilized in scientific research, education, and governmental decision-making. Access to the CGSS data requires registration and application through the official system, with approval needed to obtain the data. For further information or to request access, please contact the CGSS data management team at http://cgss.ruc.edu.cn/.

## Footnotes

^1^Detailed information is provided in Supplementary Table S1.

^2^According to the urban-rural residents' health insurance contribution standards for the past years jointly issued by the National Health Insurance Bureau, the Ministry of Finance and the State Administration of Taxation, from 2015 to 2022, the minimum standard of individual contributions for rural residents to participate in the basic social health insurance increased from 200 yuan in 2015 to 350 yuan in 2022.

^3^According to national and provincial policies, “prescription limit management is implemented for outpatient chronic diseases, generally prescribing medications for 0.5-1 month at a time, with a maximum of 3 months.”
