# Supplementary material for: Impact of Urban-Rural Resident Basic Medical Insurance integration on individual social fairness perceptions: evidence from rural China
Source: Front Public Health. 2024 Aug 29;12:1408146. doi: 10.3389/fpubh.2024.1408146 (PMC11390400; doi:10.3389/fpubh.2024.1408146)
Supplement: Supplementary file 1 [file Table_1.docx]

Supplementary Material

Table S1: Integration Timeline and Methods of URRBMI in Pilot City

| **Number** | **City Name** | **Integration Year** | **Integration Method** |
| --- | --- | --- | --- |
| 1 | Chongqing | 2009 | One System, Multiple Standards |
| 2 | Chengdu | 2009 | One System, Multiple Standards |
| 3 | Tianjin | 2010 | One System, Multiple Standards |
| 4 | Hangzhou | 2011 | One System, Multiple Standards |
| 5 | Leshan | 2011 | One System, Multiple Standards |
| 6 | Shantou | 2011 | One System, Multiple Standards |
| 7 | Wuzhong | 2012 | One System, One Standard |
| 8 | Yan'an | 2012 | One System, Multiple Standards |
| 9 | Kunming | 2013 | One System, One Standard |
| 10 | Sanming | 2013 | One System, One Standard |
| 11 | Luzhou | 2015 | One System, Multiple Standards |
| 12 | Meishan | 2015 | One System, Multiple Standards |
| 13 | Linyi | 2015 | One System, One Standard |
| 14 | Rizhao | 2015 | One System, One Standard |
| 15 | Tai'an | 2015 | One System, Multiple Standards |
| 16 | Jining | 2015 | One System, One Standard |
| 17 | Yantai | 2015 | One System, Multiple Standards |
| 18 | Guangzhou | 2015 | One System, One Standard |
| 19 | Huzhou | 2015 | One System, One Standard |
| 20 | Xining | 2015 | One System, One Standard |

Note: Since the CGSS data is available only up to the year 2015, this table includes only the cities that implemented integration before 2015.
